# Comparative transcriptome analysis of field- and chamber-grown samples of *Colobanthus quitensis* (Kunth) Bartl, an Antarctic flowering plant

**DOI:** 10.1038/s41598-018-29335-4

**Published:** 2018-07-23

**Authors:** Sung Mi Cho, Hyoungseok Lee, Hojin Jo, Horim Lee, Yoonjee Kang, Hyun Park, Jungeun Lee

**Affiliations:** 1 Unit of Polar Genomics, Korea Polar Research Institute, KIOST, Incheon, 21990 Republic of Korea; 2 Polar Science, University of Science and Technology, Incheon, 21990 Republic of Korea; 3 Department of Biotechnology, Duksung Women’s University, Seoul, 01369 Republic of Korea

## Abstract

*Colobanthus quitensis* is one of the two vascular plants inhabiting the Antarctic. In natural habitats, it grows in the form of a cushion or mats, commonly observed in high latitudes or alpine vegetation. Although this species has been investigated over many years to study its geographical distribution and physiological adaptations to climate change, very limited genetic information is available. The high-throughput sequencing with a *de novo* assembly analysis yielded 47,070 contigs with blast-hits. Through the functional classification and enrichment analysis, we identified that photosynthesis and phenylpropanoid pathway genes show differential expression depending on the habitat environment. We found that the known ‘plant core environmental stress response (PCESR)’ genes were abundantly expressed in Antarctic samples, and confirmed that their expression is mainly induced by low-temperature. In addition, we suggest that differential expression of thermomorphogenesis-related genes may contribute to phenotypic plasticity of the plant, for instance, displaying a cushion-like phenotype to adapt to harsh environments.

## Introduction

Land plants are susceptible to adverse environmental conditions. Extreme temperatures, drought, high salinity, and high UV radiation are typical environmental stressors that can damage cellular structures and impair physiological function^1^, leading to inhibited photosynthesis, retarded growth and reduced yields in plants^2,3^. To cope with these environmental stressors, plants developed stress resistance strategies, including intracellular physiological and metabolic changes such as increasing membrane fluidity and expression of cytoprotective metabolites by regulating stress signal transduction^1,3,4^. On the other hand, plants are exposed to environmental fluctuations that can drive phenotypic plasticity in plants. Phenotypic plasticity has been defined as the ability of an individual organism to alter its physiology/morphology in response to changes in environmental conditions^5^. Since the range of phenotypic plasticity varies depending on the species, it can cause different responses to overcome environmental stress and affect crop yields and plant distribution^6^.

Perennial plants growing in a cushion form commonly found in high latitudes or alpine vegetation are generally round hemispherical or mat shaped^7^. In extreme ecosystems, these growth forms are thought to play an ecological role in minimizing the loss of heat and moisture which not only helps a plant survive but also creates a microclimate shelter for maintaining species diversity of microbes, insects, or other young plants^8,9^. In addition, several hundreds of compact cushion plants spanning to various families and genera including *Silene acaulis* (Caryophyllaceae), *Azorella compacta* (Apiaceae), *Androsace helvetica* (Primulaceae), and *Raoulia eximia* (Asteraceae), are considered to be good examples of convergent evolution related to the phenotypic plasticity to cope with extreme environments^7^.

*Colobanthus quitensis* (Caryophyllaceae) is a self-fertilizing species that grows in the form of a cushion or mats. It is distributed broadly from Mexico (17°N) to the Antarctic Peninsula (68°S) and at altitudes ranging from 0 to 4200 meters^10–12^ and is known to exhibit considerable morphological variation depending on their habitats^10–14^. The morphology analysis of the *C. quitensis* populations along an Antarctic-subantarctic latitudinal gradient revealed that populations at higher latitudes have smaller and thicker leaves with higher mesophyll thickness, narrower adaxial surfaces, increased pigments and reduced epidermis^15,16^.

Structural and physiological characteristics responsible for the high tolerance of *C. quitensis* to extreme conditions were described in terms of the difference in leaf anatomical traits and ultracellular structures and varied photosynthetic responses^16–22^. In laboratory controlled conditions, the leaves of *C. quitensis* had anatomical and physiological changes when exposed to abiotic stressors such as low-temperature, high light or ultraviolet light^18–21,23^ and the photosynthetic efficiency was primarily regulated by temperature^21,23^. In addition, field studies have shown that the OTC (Open Top Chamber) warming effects affect plant growth by influencing plant morphoanatomical traits, cellular chemical composition, and photosynthetic parameters^17,22^. These suggest that the developmental changes induced by temperature ultimately determine photosynthetic efficiency.

On the other hand, the adaptation of Antarctic *C. quitensis* to the extreme environment has been explained by population-specific genetic evolution^18–20^. The studies with two geographically separated populations have shown that the Antarctic ecotype of *C. quitensis* undergoes less photoinhibition than an alpine ecotype because it has better recovery rates of PSII than the alpine ecotype^19,20^. This suggests that the Antarctic ecotype of *C. quitensis* might have developed a unique stress response mechanism operating at the molecular level to survive in a harsh environment.

Despite previous studies on morphological differences and stress resistance of *C. quitensis*, there is no comprehensive report available on genetic regulation using plants growing in a natural habitat. To elucidate the regulatory networks involved in gene expression associated with environmental stress-driven tolerance responses and morphological plasticity of *C. quitensis*, we constructed a *de-novo* transcriptome assembly and compared the transcriptome profiles on plant samples collected from low-temperature environment of Antarctic natural habitat versus those grown with the mild growth condition in the laboratory. We classified the genes according to functional categories through GO and KEGG analysis, identified genes that differ in their expression level with the habitat, and deduced their potential functions through ortholog analysis with the model plant. Our findings may provide new insights into the genetic control system developed by plants adapted to the extreme environment.

## Results

### Morphology of ANT and LAB samples of *C. quitensis*

**Figure 1:**
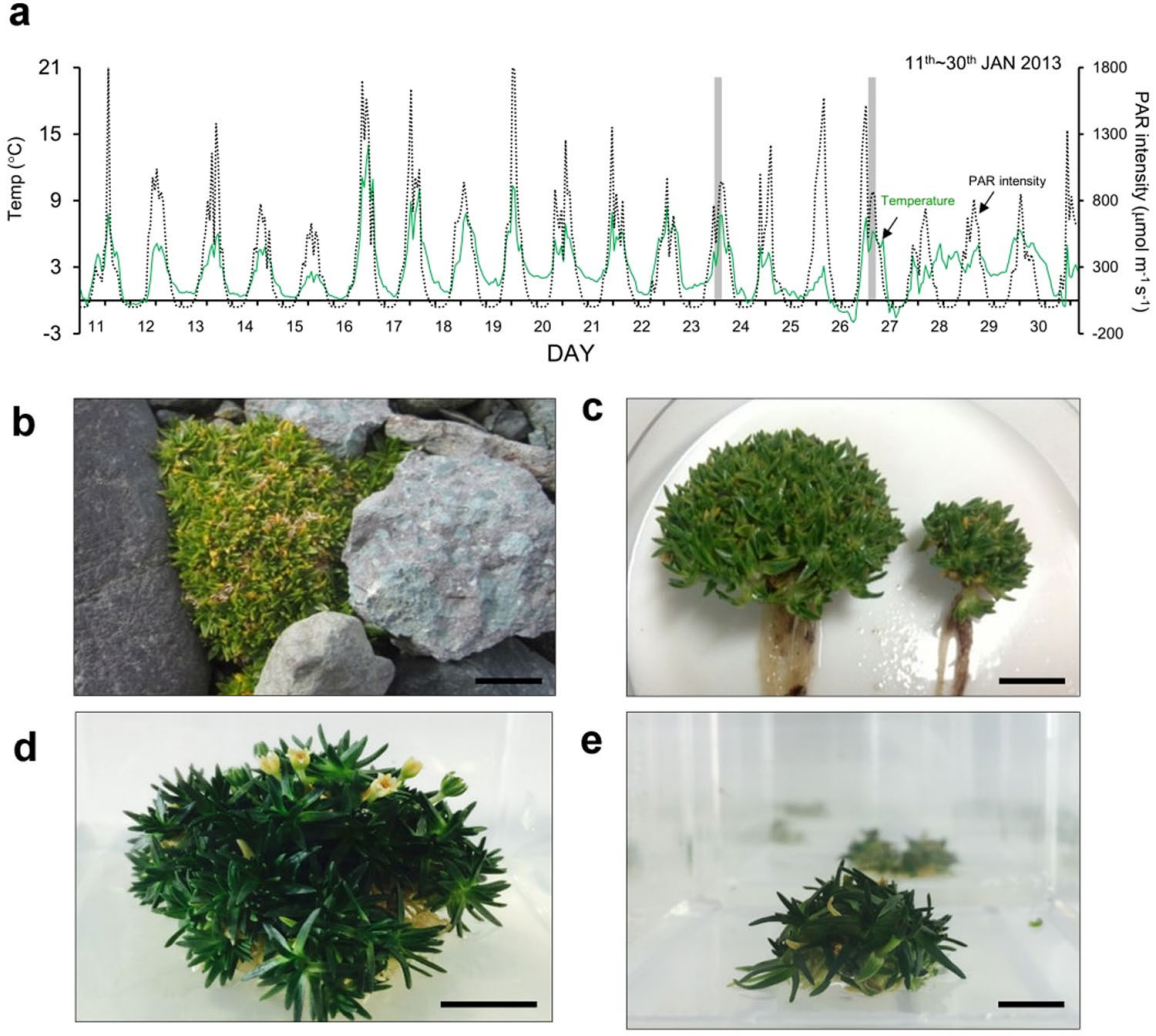
Microclimates of the sampling site in Antarctica and morphology of *C. quitensis* plants (**a**) The temperature (green solid line) and light intensity (black dotted line) of the plant sampling sites had been measured during summer research activities in January 2013. The temperature of the site was measured from a temperature sensor installed 5 cm above the soil surface. During the period, the average temperature was 4.3 °C and 1.8 °C for day and night, respectively. Luminosity was close to zero from midnight to 04:00 AM. For ANT samples, plants were harvested between 11:00 and 13:00 during mid-to-late January (shaded). (**b,c**) Wild plants from the natural habitat of the Antarctic, and (**d,e**) a plant grown in a climate chamber. Side-bars indicate 1 cm. The *C. quitensis* found on the coast of the Baton Peninsula are often found around rocks and their overall shape is hemispherical or similar to moss carpets. When young plants with a diameter of 2 cm or less were transferred to a climate chamber maintained at a temperature of 16 °C and grown in nutrient medium, the density of leaves became much looser than those of similar sized Antarctic plants.

*C. quitensis* plants have shown changes in morphology and physiological responses with respect to geographical distribution and microclimate differences^18,20,24^. The transcriptome profile of extremophile plants in their natural habitats provides biological information as to how they optimize gene regulation to overcome environmental stresses. Therefore, to investigate the molecular and genetic mechanisms associated with stress tolerance and morphological plasticity due to environmental stress in *C. quitensis*, we compared the transcriptome profiles between plants inhabiting in Antarctic Barton Peninsula and plants cultivated in the mild growth condition in the laboratory. The Antarctic field plants designated ANT were collected in the Baton Peninsula in January 2013 (Fig. 1a). The laboratory cultures designated as LAB were cultivated at 16 °C, a temperature within the known optimal leaf temperature range (14~18 °C) for photosynthesis of *C. quitensis*^21^. The morphological differences between the LAB and ANT samples were observed during growth. While the LAB plants have longer leaf blades and elongated hypocotyls, the ANT plants have a denser structure consisting of short and thick leaves (Fig. 1b–d).

### Deep sequencing, *de novo* assembly, and functional annotation of the *C. quitensis* transcriptome

Total 160,264,674 reads were generated from Illumina HiSeq. 2500 run. Trimming of raw sequences provided 143,972,486 high-quality reads (Q > 30) comprising 13.7 Gb of sequences. Using CLC Genome Workbench assembly module, the reads were assembled into 101,690 contigs averaging 764 bp and N50 value of 1,058 bp. The assembled contigs were clustered into 95,010 representative contigs using the CD-HIT-EST program (see Supplementary Table S1).

To annotate the assembled unigenes, the sequences were blast searched against GenBank nr or UniProt databases using the BLASTX algorithm. When we blasted them against the nr database, 47,070 contigs had at least one significant alignment to an existing protein data with an E-value of 1 × 10^−5^ and 36,387 unigenes (38.3%) had mapping results (Supplementary Table S1). When searched in UniProt databases, 45,037 genes have orthologs (47.4%) with existing protein sequences in the database. Among those, 10,456 genes were predicted as complete or putative complete genes (11%), 11,837 genes were predicted as putative new genes (12.5%) and 38,116 genes remained as unknown (40.1%) (Supplementary Table S1). The species distribution of BLAST matches to the UniProt plant databases is shown in Supplementary Fig. S1. The majority of the genes were matched to the dicot genes, predominantly with *A. thaliana* (42%) and *Vitis vinifera* (11%).

To classify the functions of the predicted genes of *C. quitensis*, GO terms were assigned to total contigs. Based on classification of GO terms, a total of 26,346 contigs were assigned with at least one GO term and classified into different categories using the PlantGO slim terms (Supplementary Fig. S2, Table S2). In terms of ‘biological processes’, the top 2 GO terms were ‘metabolic process’ and ‘cellular Process’. In terms of ‘molecular function’, ‘catalytic activity’ and ‘binding’ were top 2 GO terms. To infer the functions and the utilities of the genes in the biological system, total contigs were blast queried against the KEGG databases^25^ (organism code: ath) with E-value < 10^−10^. As a result, 11,950 contigs were mapped to KEGG pathways with hierarchy and they are classified into 5 functional categories (Supplementary Fig. S3). The top 5 pathways are ‘translation’, ‘carbohydrate metabolism’, ‘amino acid metabolism’, ‘folding, sorting and degradation’ and ‘energy metabolism’. For the more specific pathways of the lower hierarchy, the top 3 pathways were ‘ribosome’, ‘protein processing in endoplasmic reticulum’ and ‘oxidative phosphorylation’ (Supplementary Table S3).

### SSR Identification

Simple-sequence repeats (SSRs) are well established and have increasingly become the marker of choice for population genetic analyses due to their codominant, highly polymorphic and highly reproducible nature^26^. *C. quitensis* is an important species for studying plant ecology with geographic parameters. Studies about physiological differences involving genetic diversity have been performed among *C. quitensis* populations^18,19,27^. Given the research interests and the importance of *C. quitensis*, molecular markers for this species can be valuable resources. We identified EST-SSRs from assembled contigs during the transcriptome analysis of *C. quitensis*. A total of 8,619 distinct loci containing motifs between one and six nucleotides in size were discovered in 7,749 contigs. Approximately 60.5% of SSRs were mononucleotides, 28.3% were trinucleotides, 9.3% were dinucleotides and the remaining ~2% were tetra-, penta-, and hexanucleotides. The most frequent dinucleotide SSR was AG/CT (382/4.43%), however, for trinucleotide SSRs, AAC/GTT, AAT/ATT, ACC/GGT, AAG/CTT, and ATC/ATG show similar frequency in the range of 4.2–4.9% (Supplementary Table S4).

### Identification of differentially expressed genes in ANT vs. LAB samples

**Figure 2:**
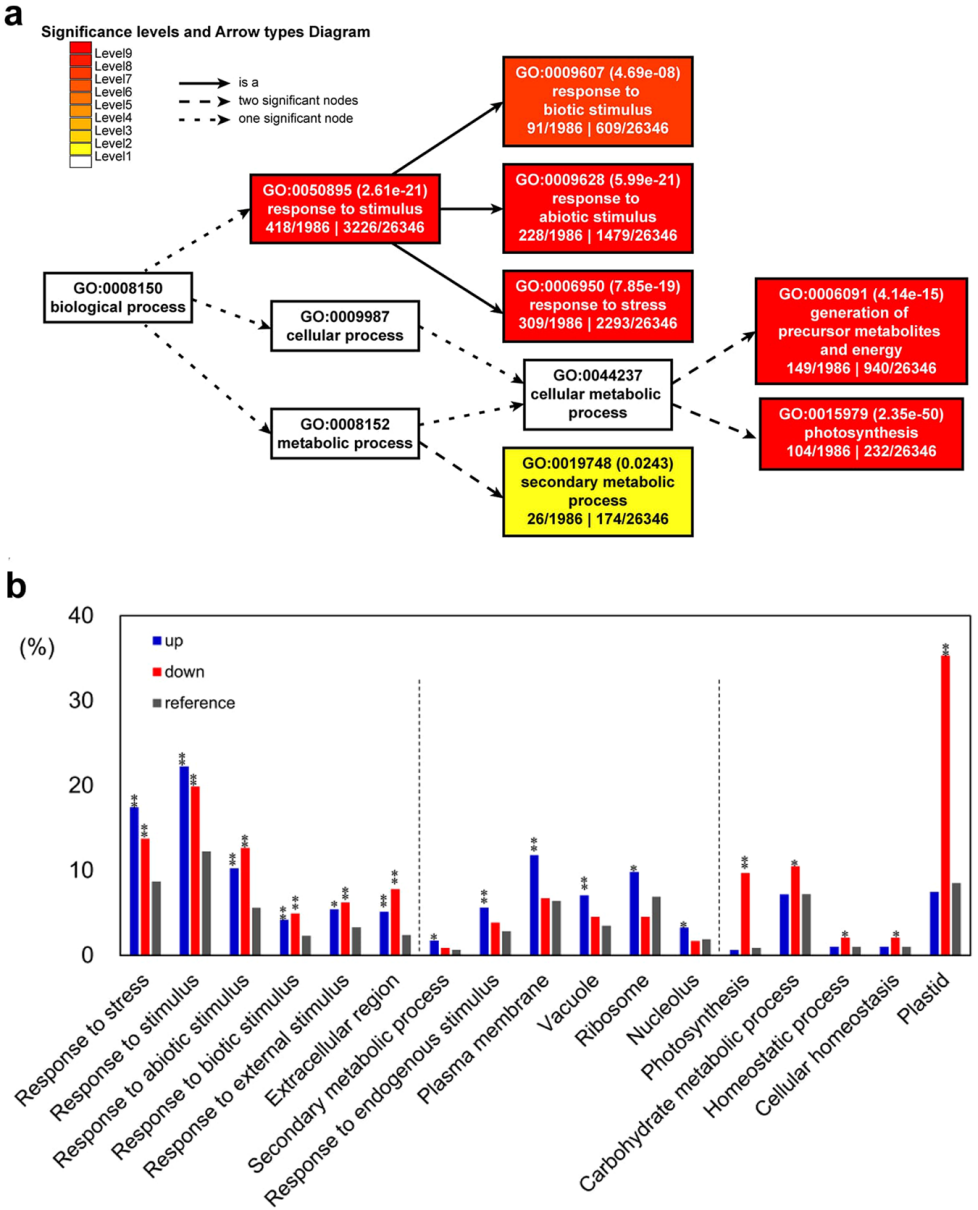
Functional classification of DEGs by plant GO slim terms. (**a**) GO terms of all DEGs with significance (*FDR* < 0.05, Fisher’s exact test) are presented with box colors when compared with those of a total of 26,346 GO-annotated contigs. The figure was drawn by AgriGO analysis tool^65^ (**b**) The percentage of enriched GO terms of upregulated- and downregulated gene groups were compared with those of a total of contigs. The blue, red, and grey bars indicate the percentage of GO terms of the upregulated, downregulated and a total of contigs, respectively. Statistical significance is indicated with asterisks (^*^FDR < 0.05, ^**^FDR < 0.001).

To compare the transcriptome of ANT and LAB samples, we remapped the reads generated from each library to the assembled 95,010 contigs and counted the read numbers that mapped to each contig. Statistical analysis revealed totally 3,902 differentially expressed genes (DEGs) in which 2,127 transcripts were upregulated and 1,775 transcripts were downregulated in ANT samples compared to LAB sample with a cutoff of *FDR* corrected *p*-value < 0.05 (beta-binomial test) (Supplementary Tables S5 and S6). Comparative GO enrichment analysis was conducted on the subsets of 3,900 DEGs, 2,127 upregulated DEGs, and 1,775 downregulated DEGs, respectively with the complete set of transcripts sets, with Fisher’s exact test (*FDR* < 0.05). Among the GO terms included in ‘biological process’ category, the ‘response to stress (GO: 0006950)’ and the ‘photosynthesis (GO: 0015979)’ categories were the most enriched GO terms in the differentially expressed gene group (Fig. 2a). To elaborate, GO terms of ‘response to stress’, ‘response to (abiotic, biotic or external) stimulus’ and ‘extracellular region’ were significantly enriched in both upregulated and downregulated gene subsets. In ANT compared to LAB transcriptomes, however, the GO terms of ‘secondary metabolic process’, ‘response to endogenous stimulus’, ‘plasma membrane’, ‘vacuole’, ‘ribosome’ and ‘nucleolus’ and the GO terms of ‘photosynthesis’ and ‘carbohydrate metabolic process’, ‘cellular homeostasis’ and ‘plastid’ were enriched only in the upregulated and downregulated gene subsets, respectively (Fig. 2b and Supplementary Tables S7 and S8).

### Differentially altered metabolic pathways in ANT samples

**Figure 3:**
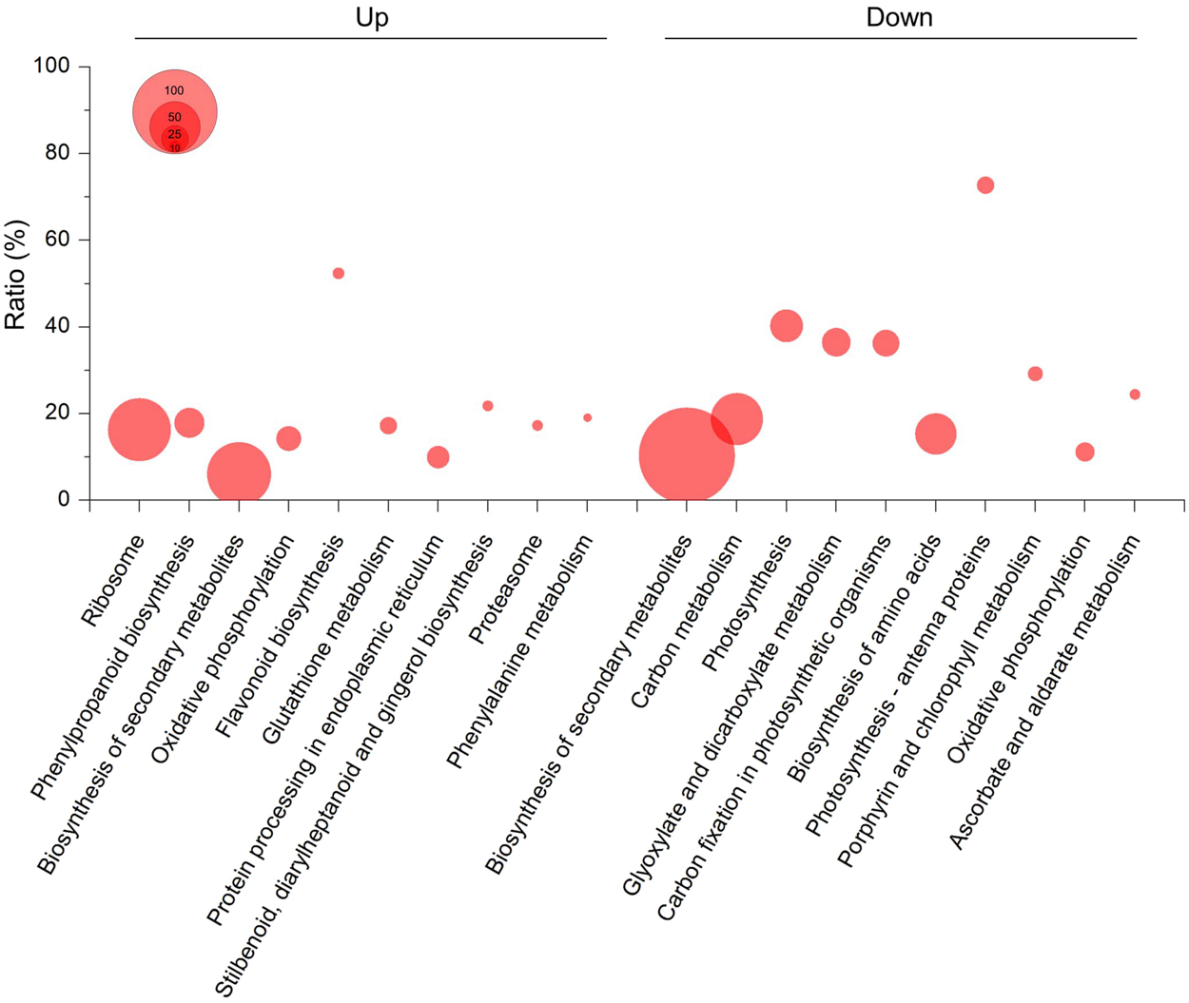
A scatterplot of enriched KEGG pathways in upregulated- and downregulated gene groups. The significantly enriched KEGG pathways were indicated as dots (*FDR* < 0.05, Fisher’s exact test) and the dot sizes represent the number of the genes included in each cluster. The horizontal axis represents the ratio of the number of differentially expressed genes and the number of all genes in the pathway.

To determine the altered metabolic pathways in ANT samples, a KEGG enrichment analysis was performed on DEGs in ANT vs. LAB transcriptomes. Among the statistically enriched metabolic pathways (*FDR* < 0.01, Fisher’s exact test) (Supplementary Tables S9 and S10), the top 10 enriched KEGG pathways of upregulated and downregulated genes are shown in Fig. 3. The ‘ribosome’, ‘phenylpropanoid biosynthesis’, ‘biosynthesis of secondary metabolites’, ‘oxidative phosphorylation’, ‘flavonoid biosynthesis’, ‘glutathione metabolism’, etc. were identified as the enriched pathways in the upregulated genes, whereas ‘biosynthesis of secondary metabolites’, ‘carbon metabolism’, ‘photosynthesis’, etc. were identified as the enriched pathways in the downregulated genes (Fig. 3 and Supplementary Tables S7–S10). KEGG and GO enrichment analyses on DEGs suggested that *C. quitensis* exhibits different cellular performance in ANT vs. LAB conditions by regulating genes in various metabolic pathways.

Regarding the ‘photosynthesis’ pathway, it is noteworthy that putative *PGR5* (*PROTON GRADIENT REGULATION 5*, Contig18861) gene, known as an essential protein for photoprotection^28,29^, was upregulated, while many photosynthesis-related genes were downregulated in ANT samples (Supplementary Tables S7–S10).

**Figure 4:**
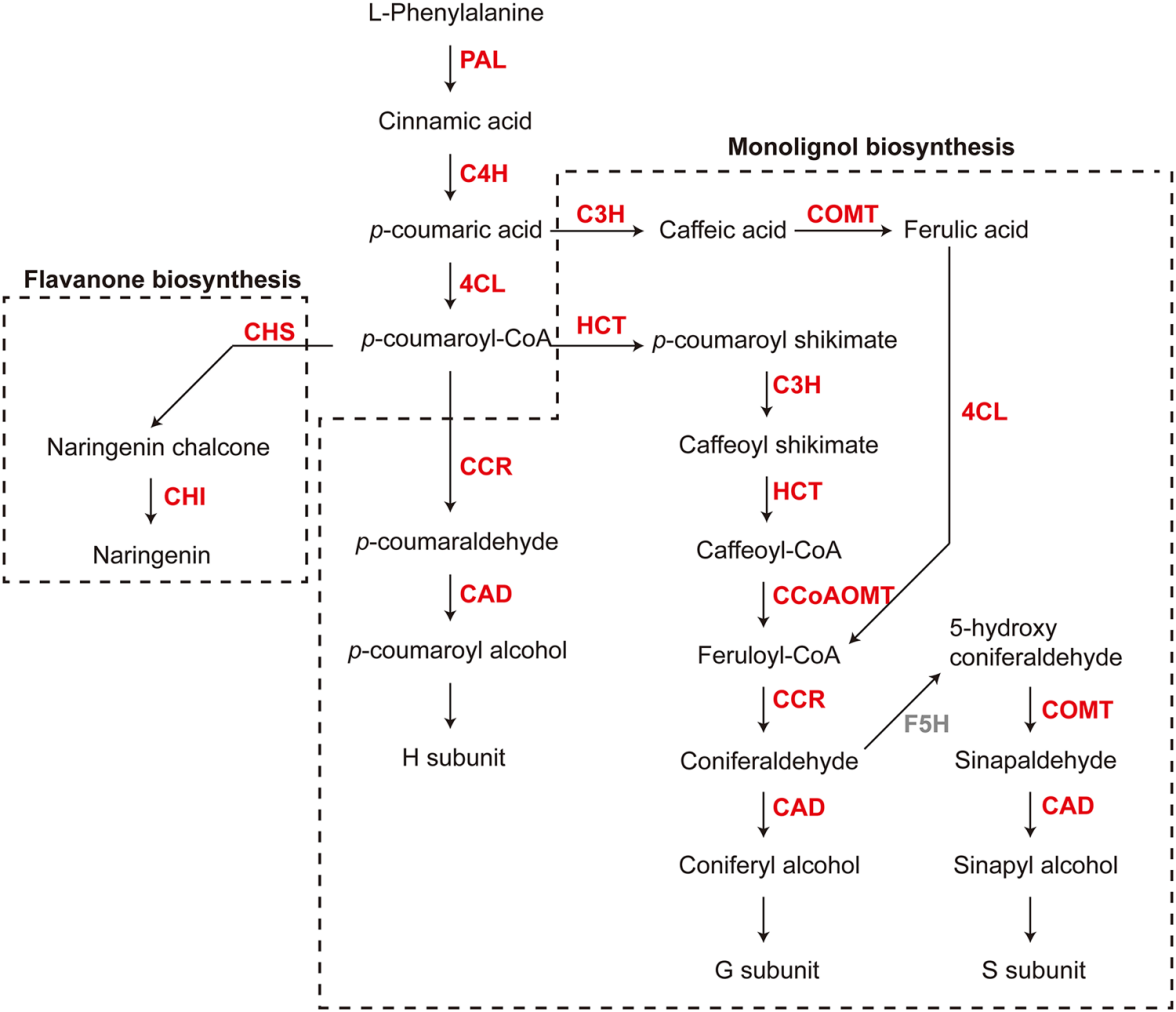
The genes of ‘phenylpropanoid biosynthesis’ pathway were upregulated in ANT samples. The ‘phenylpropanoid biosynthesis’ pathway consists of the ‘monolignol biosynthesis’ (KEGG ID: M0039) and the ‘flavanone biosynthesis’ (KEGG ID: M00137) pathways, which convert phenylalanine to lignin and naringenin, respectively. The major enzymes in these pathways tended to be upregulated in ANT samples, and these are marked in red.

We also found that the ‘phenylpropanoid biosynthesis’ (KEGG ID: map00940) pathway genes were considerably enriched in the upregulated gene group in ANT sample (*p*-value: 1.7 × 10^−19^), (Figs 3 and 4 and Supplementary Table S9). In plants, phenylpropanoids are a group of secondary metabolites derived from phenylalanine and have a variety of functions as structural and signaling molecules^30^. The major modules of ‘phenylpropanoid biosynthesis’ are the ‘monolignol biosynthesis’ (KEGG ID: M0039) and the ‘flavanone biosynthesis’ (KEGG ID: M00137), which convert phenylalanine to lignin and naringenin, respectively. A total of 28 contigs in the ‘monolignol biosynthesis’ pathway were found to be increased in ANT samples (Table 1), and they include (1) Phenylalanine ammonia-lyase (PAL; Contig20542), cinnamic acid 4-hydroxylase (C4H; Contig49114, Contig70533) and 4-hydroxycinnamoyl-CoA ligase (4CL; Contig3499), enzymes that play an important role in the formation of the substrate *p*-coumaroyl-CoA in both monolignol and flavanone biosynthesis, (2) hydroxycinnamoyl-CoA shikimate hydroxycinnamoyl transferase (HCT; Contig13431, Contig29449, Contig 40563) enzymes that function in a branched pathway, and 3) hydroxycinnamoyl-CoA reductase (CCR; Contig33804) and cinnamyl alcohol dehydrogenase (CAD; Contig24021) enzymes that form lignin monomers. On the other hand, only two enzymes in the ‘flavanone biosynthesis’ pathway, chalcone synthase (CHS; Contig2685) and chalcone isomerase (CHI; Contig16136) enzymes, showed an increase in expression in ANT samples (Fig. 4 and Table 1).

**Table 1:** Expression of *C. quitensis* genes involved in phenylpropanoid biosynthesis pathway.

Contig ID	Arabidopsis ortholog	Genes^*^	EC	FPKM		*p-value*
				**LAB**	**ANT**	
Contig20542	AT2G37040	PAL1	EC:4.3.1.24	26.7	131.3	1.6 × 10^−13^
Contig20541	AT2G37040	PAL1	EC:4.3.1.24	8.5	31.7	4.2 × 10^−4^
Contig49114	AT2G30490	C4H	EC:1.14.13.11	2.4	15.8	1.7 × 10^−3^
Contig70533	AT2G30490	C4H	EC:1.14.13.11	1.9	18.9	2.4 × 10^−4^
Contig22559	AT2G40890	CYP98A3	EC:1.14.13.36	17.6	48.4	6.3 × 10^−4^
Contig24607	AT2G40890	CYP98A3	EC:1.14.13.36	16.6	49.2	2.4 × 10^−4^
Contig33804	AT1G80820	CCR2	EC:1.2.1.44	73.4	229.9	4.4 × 10^−13^
Contig7537	AT1G67980	CCoAMT	EC:2.1.1.104	39.7	141.8	1.1 × 10^−10^
Contig16972	AT4G34050	CCoAOMT1	EC:2.1.1.104	118.7	424.2	0
Contig28757	AT5G54160	OMT1	EC:2.1.1.68	854.4	1773.0	0
Contig40563	AT5G48930	HCT	EC:2.3.1.133	12.2	42.2	1.4 × 10^−4^
Contig29449	AT5G48930	HCT	EC:2.3.1.133	22.7	80.4	3.8 × 10^−7^
Contig13431	AT5G48930	HCT	EC:2.3.1.133	15.1	76.2	3.9 × 10^−9^
Contig24021	AT1G72680	CAD1	EC:1.1.1.195	94.1	345.6	0
Contig25236	AT4G37990	CAD8	EC:1.1.1.195	24.5	88.0	1.3 × 10^−7^
Contig3499	AT1G62940	4CL	EC:6.2.1.12	3.2	49.5	9.6 × 10^−10^
Contig24005	AT1G68850	PRX	EC:1.11.1.7	9.5	40.8	2.7 × 10^−5^
Contig21838	AT1G71695	PRX	EC:1.11.1.7	46.1	179.7	2.7 × 10^−14^
Contig28685	AT2G38380	PRX	EC:1.11.1.7	11.7	46.6	1.7 × 10^−5^
Contig68730	AT4G21960	PRX	EC:1.11.1.7	46.2	120.3	5.0 × 10^−6^
Contig24815	AT4G21960	PRX	EC:1.11.1.7	212.8	377.8	1.3 × 10^−5^
Contig16077	AT4G37520	PRX	EC:1.11.1.7	1.2	24.1	9.2 × 10^−6^
Contig18878	AT5G05340	PRX	EC:1.11.1.7	9.3	52.3	2.0 × 10^−7^
Contig86217	AT5G05340	PRX	EC:1.11.1.7	13.9	73.4	3.7 × 10^−9^
Contig66598	AT5G05340	PRX	EC:1.11.1.7	33.2	92.9	8.6 × 10^−6^
Contig59359	AT5G22410	PRX	EC:1.11.1.7	175.6	319.5	3.4 × 10^−5^
Contig31962	AT5G66390	PRX	EC:1.11.1.7	9.4	105.6	0
Contig33299	AT5G66390	PRX	EC:1.11.1.7	11.1	101.3	1.8 × 10^−15^
Contig2685	AT5G13930	CHS	EC:2.3.1.74	8.7	67.7	0
Contig16136	AT3G55120	CHI	EC:5.5.1.6	36.8	80.0	0

PAL: phenylalanine ammonia lyase; C4H: cinnamic acid 4-hydroxylase; 4CL: 4-hydroxycinnamoyl-CoA ligase; CYP98A3: Cytochrome P450 98A3 (Coumaroyl shikimate 3′-monooxygenase); HCT: hydroxycinnamoyl-CoA:shikimate hydroxycinnamoyl transferase; C3H: p-coumaroyl shikimate 3′-hydroxylase; CCoAOMT: caffeoyl-CoA O-methyltransferase; CCR: hydroxycinnamoyl-CoA reductase; F5H: ferulic acid 5-hydroxylase; COMT: caffeic acid/5-hydroxyferulic acid O-methyltransferase; CAD: cinnamyl alcohol dehydrogenase; PRX: peroxidase; CHS: chalcone synthase; CHI: chalcone isomerase.

### The increased expression of the ‘plant core stress responsive genes’ in ANT samples

When plants are treated with abiotic stress, a group of stress genes is commonly expressed at the early stage. This gene response is called ‘plant core environmental stress response (PCESR)’, which is well established in *Arabidopsis thaliana*^31,32^. PCESR is not limited to Arabidopsis but is also found in other plant species, suggesting that PCESR to various abiotic stimuli is conserved in plants^31,33–35^. We hypothesized that the plants growing in Antarctic fields would display high expression of abiotic stress tolerance genes to cope with the extreme environmental stress. Among the 8530 reciprocal-best-hit genes between two species (Supplementary Fig. S4), 35 *C. quitensis* orthologs to the Arabidopsis PCESR genes were found (Table S11), and their expression was compared in LAB and ANT samples. As a result, about 40% (17/35) of the PCESR orthologs showed significantly high levels of transcripts (*p* < 0.05) in ANT samples (Table 2).

**Table 2:** High expression of PCESR orthologs in ANT sample.

Contig ID	Arabidopsis ortholog	Description	FPKM		*p-*value
			LAB	ANT	
Contig22074	AT1G27730	Zinc finger protein ZAT10-like	11.7	48.5	8.8 × 10^−6^
Contig28814	AT5G59820	Zinc finger protein ZAT12-like	2.4	20.7	1.9 × 10^−4^
Contig38763	AT1G76650	Calcium-binding protein CML37	0.4	44.2	2.8 × 10^−10^
Contig14494	AT4G17500	AP2 like transcription factor	8.2	54.5	2.7 × 10^−8^
Contig35858	AT1G69490	NAC transcription factor 027	10.0	27.0	7.0 × 10^−3^
Contig16997	AT4G37260	MYB44-like transcription factor	6.0	19.9	7.0 × 10^−3^
Contig45609	AT3G44260	CCR4-NOT transcription complex family protein	0.6	15.3	3.0 × 10^−4^
Contig7995	AT2G44500	DUF246 domain- protein	2.5	9.7	3.4 × 10^−2^
Contig21564	AT5G26340	Hexose transporter	81	138	3.0 × 10^−2^
Contig99760	AT2G35930	E3 ligase PUB23-like	0.6	6.6	2.8 × 10^−2^
Contig15740	AT5G10190	Major facilitator superfamily	12.0	32.1	4.2 × 10^−3^
Contig34911	AT3G16720	Ring finger protein	2.8	13.0	9.6 × 10^−3^
Contig20878	AT3G05200	Ring finger family protein	9.7	25.9	8.3 × 10^−3^
Contig42834	AT5G06860	Polygalacturonase inhibiting protein	22.5	125.3	2.3 × 10^−14^
Contig21412	AT5G45340	ABA8 -hydroxylase CYP707A1	2.0	39.8	1.8 × 10^−8^
Contig_67594	AT5G64660	U-box domain protein 27	5.8	16.9	2.0 × 10^−2^
Contig13526	AT4G24570	Mitochondrial uncoupling protein 5	28.0	93.4	2.6 × 10^−7^

**Figure 5:**
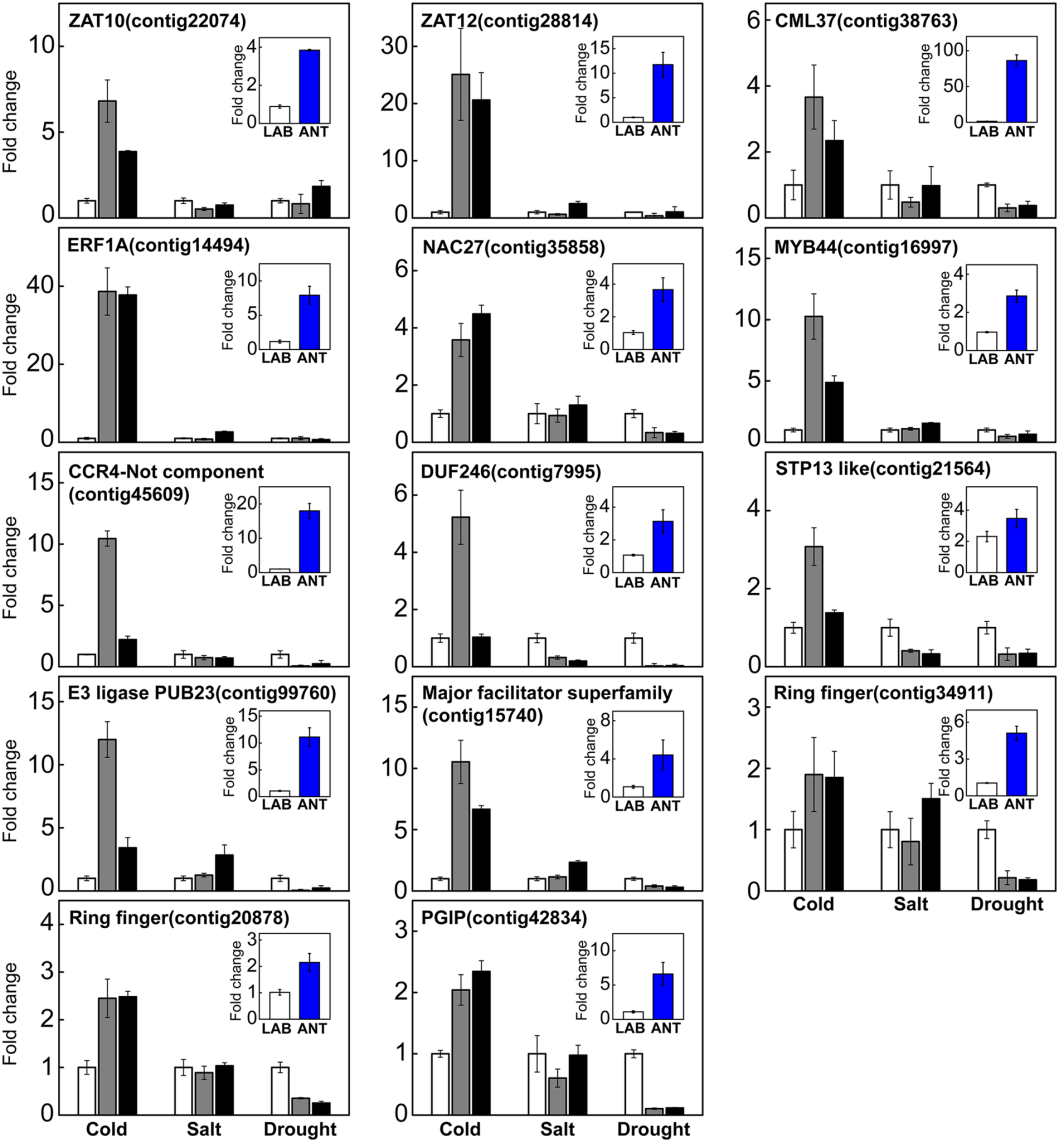
qPCR analysis of PCESR - orthologs from *C. quitensis* in LAB- vs. ANT samples and the plants exposed to different abiotic stressors. The main graphs are the results of the abiotic stress treated samples and inbox graphs are the results of the LAB- vs. ANT samples. The genes were selected from the 14 PCESR orthologs which have higher expression values in ANT samples from RNA-Seq results. For stress treatment, plants at similar developmental stages were treated with low-temperature (2 °C), high salt (150 mM NaCl) or dehydration for 1 and 7 days. The left vertical axis indicates the relative ratio of transcript abundance of selected genes compared to *18S rRNA* and *TIM* (Contig19814) which were predicted as the best internal controls by reference gene prediction tools^71,72^. Mean and standard deviation are presented (n = 3) and white, grey and black bars display the 0, 1 and 7 days of stress treatments, respectively. The primer sequences used in qPCR analysis are listed in Supplementary Table S12.

We verified their expression levels by quantitative RT-PCR (qPCR) and confirmed if the observed levels are induced by abiotic stimuli including low-temperature, salinity, or drought. Plants were subjected to each abiotic stress for 24 hours and the RNA expression of the genes between treated and non-treated samples was compared. Among the genes tested, 14/17 showed higher expression levels in ANT samples and were induced by abiotic stresses (Fig. 5). These included the genes with various biological functions such as transcription factors including an APETALATA2 (AP2)/ethylene response factor (ERF) protein (Contig14494), a NAC27-like (Contig38763), a MYB44-like protein Contig16997); C2H2 type zinc finger proteins including ZAT10 (Contig22074) and ZAT12 (Contig28814); a CCR4-associated factor (Contig45609); a calcium-binding protein (Contig38763); a DUF246-containing protein (Contig7995); a sugar transporter 13 (STP13)-like gene (Contig21564); a PUB23 E3 ligase family protein (Contig99760); a major facilitator superfamily gene (Contig15740); ring finger proteins (Contig34911, Contig20878); and a polygalacturonase inhibitor protein (Contig42834). It is noteworthy that these genes respond more specifically to low-temperatures among various abiotic stressors, suggesting that the temperature is the most important factor to induce stress signaling in plants.

### Transcription factors associated with morphological changes in different conditions

*C. quitensis* is a representative cushion plant, which forms a dense hemisphere with short leaves, inhabiting the alpine and high latitude. When the field plants were transferred and grown in a 16 °C climate chamber, we noticed that the compact structure of *C. quitensis* loosened and the leaves elongated (Fig. 1c,d). Recent reports have shown that some PHYTOCHROME INTERACTING FACTORS (PIFs), bHLH transcription factors, are associated with thermomorphogenesis^36–39^. In particular, in the case of Arabidopsis *PIF4* (*AT2G43010*), gene expression increases with increasing temperature, while gene expression decreases with decreasing temperature^36^. The length of hypocotyl and leaf petiole was longer than that of WT when these genes were overexpressed, and the opposite when they were knocked out^36,38,39^. These expression patterns and phenotype of transgenic plants suggest that they participate in leaf elongation caused by change in temperature^36,39,40^.

**Figure 6:**
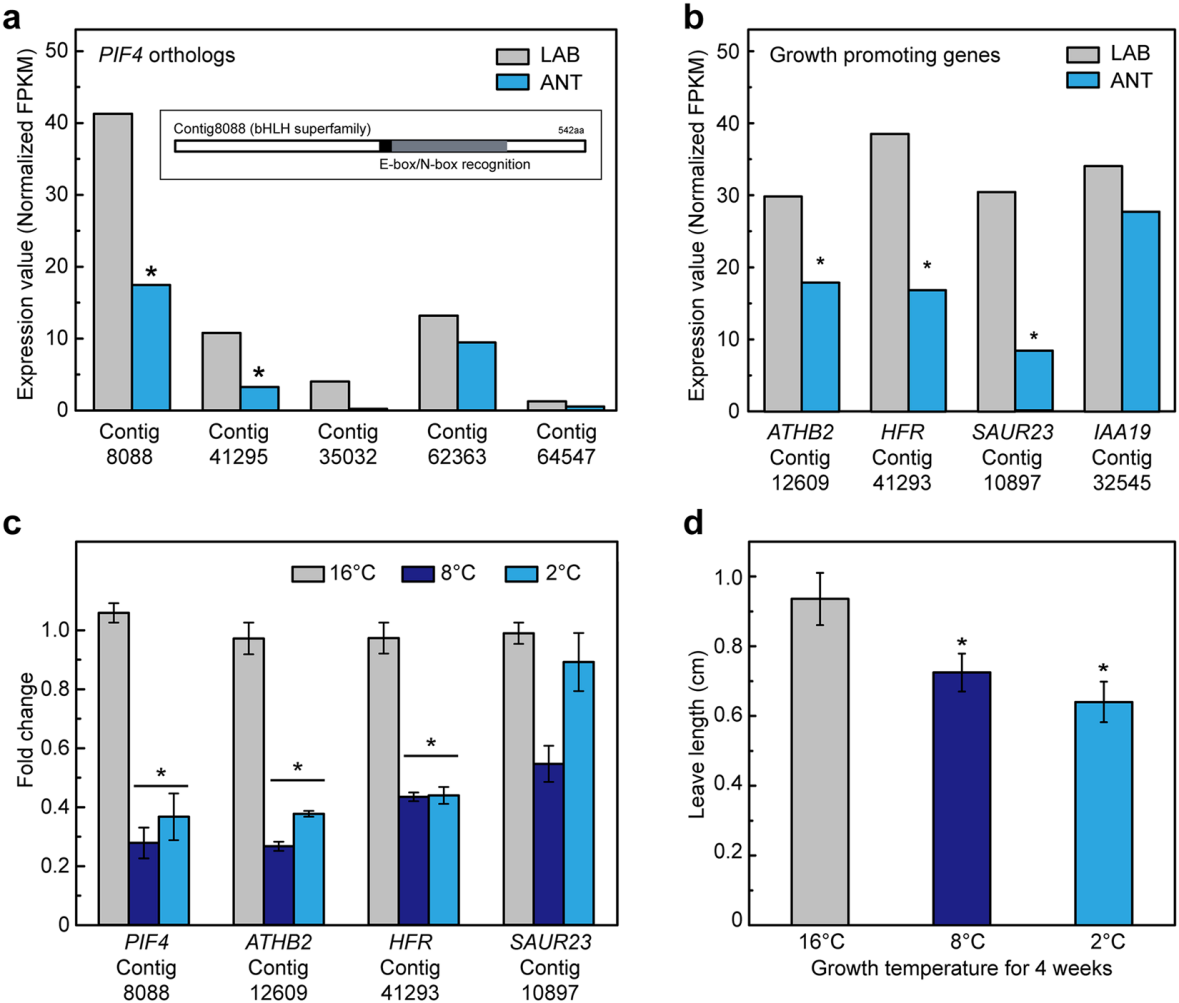
The relative gene expression of the orthologs of PIF4 and growth promoting genes as PIF4-targets. (**a**) The relative gene expression of PIF4-like orthologs which were found by TBLASTN search using PIF4 as a query. In an inbox, it is shown that Contig8088 contains the E-box/N-box recognition site. (**b**) The expression values of growth-promoting genes, *HFR*, *SAUR23*, *ATHB2*, and *IAA19* as PIF-targets. The grey and light-blue bars indicate the expression values of LAB and ANT samples, respectively. The left axis indicates the expression values (normalized FPKM). (**c**) Relative expression of *PIF4* and growth promoting genes, *ATHB2, HFR* and *SAUR23* from *C. quitensis* plants treated 2 °C, 8 °C and 16 °C for 4 weeks. Mean and standard deviations are shown (n = 3). (**d**) The leaf length of *C. quitensis* plants treated 2 °C, 8 °C and 16 °C for 4 weeks were measured by ImageJ program. Mean and standard deviations are displayed (n = 5). The significances were represented by *p*-values (^*^p < 0.05, *t*-test).

In this regard, we hypothesized that expression of *PIF4* and its associated growth-promoting genes would be decreased in ANT samples that have smaller leaves. To prove the hypothesis, first, we tried to determine if the PIF genes of *C. quitensis* were altered in expression under field vs chamber conditions. A TBLASTN search using *Arabidopsis* PIF4 as a query yielded five hits (Contig8088, Contig41295, Contig35032, Contig62363, Contig64547) containing a bHLH DNA-binding domain with an E-box/N-box specificity site were found (E-value < 10^−10^) (Fig. 6a). As expected, the expression levels of all orthologs were lower in the ANT sample, and Contig8088 showed a significant decrease (*FDR* corrected p-value < 0.05). In *Arabidopsis*, PIF4 activates the expression of growth-promoting genes such as *HFR*, *IAA29*, *IAA19*, *ATHB*, and *SAUR23*^37,39^. Therefore, we examined whether the expression of these gene orthologs known to be targets of PIF4 differs between ANT and LAB samples of *C. quitensis*. We identified the orthologs of those genes in *C. quitensis* transcriptome, and the expression values of *ATHB2* (Contig12609), *HFR* (Contig41293), and *SAUR23* (Contig10897) were lower in the ANT than LAB samples (Fig. 6b).

These results were reproducible in low-temperature-grown samples. Relative gene expression of orthologs of *PIF4* and its downstream genes, *ATHB2* and *HFR* were significantly reduced in both plants grown at 8 °C or 2 °C (Fig. 6C). As expected, leaf length was found to be reduced significantly in low-temperature-grown samples than those grown under control temperature conditions (Fig. 6d). Additionally, proliferation of root and shoot was observed when grown at 16 °C or above than at low-temperature conditions (data not shown). Taken together, the results suggest that the characteristic phenotype of field plants is associated with downregulation of PIF4 and its downstream genes.

## Discussion

Plant growth changes due to temperature variations, hyperthermal or hypothermal, has been reported in many studies^41–44^. In the case of Arctic and Antarctic plants, there have been several reports about the phenotypic plasticity. The chilling temperatures have shown to affect the organelle ultrastructure and the organization of palisade cells in *C. quitensis* mesophyll^13,24^. The plants naturally growing in the Antarctic field and the cold-acclimated plants, both have deformed chloroplasts with multi-shaped protrusions and invaginations to increase the contact area between adjacent cell compartments^24,45^. The phenotypic plasticity is also evident in another Antarctic vascular plant, *Deschampsia antarctica*, a Poaceae flowering plant growing in Antarctic regions^46,47^. Recent *in situ* warming tests on *C. quitensis* have shown that the photosynthesis response to warming is regulated by the anatomical determinants of leaf CO_2_ transfer, which enhanced mesophyll conductance and carbon assimilation, thereby promoting higher leaf carbon gain and plant growth^17,22^. This suggests that phenotypic plasticity by temperature plays a major role in plant growth and adaptation.

In this study, we observed that the leaves of *C. quitensis* elongated when grown in a growth chamber, the dense cushion shapes of the plants found in nature were loosened. It is expected that plants grown in Alpine/polar habitats form dense cushions with short leaves to minimize heat loss. On the contrary, when the temperature rises, the density of leaves in a plant decreases with prolonged leaves, thus the CO_2_ transfer would be increased, promoting plant growth. Therefore, we suggest that there are developmental regulatory genes that cause anatomical changes of the leaf with temperature and that the PIF transcription factors play a role in this developmental change. There is growing evidence that *PIF*s act as positive regulators in cell elongation and integrate multiple signaling pathways^37^. Especially, in the case of *PIF4* that promotes cell growth, its gene expression is temperature-dependent. Although *PIF*s are well known for the mechanisms involving thermomorphogenesis associated with high temperature^36,48^, there is not much discussion of the ecological benefits derived from their regulatory roles at low-temperature. In this regard, we observed that the expression of all *PIF4* orthologs was found to be lower in the Antarctic samples and the expression of genes such as *HFR*, *IAA19*, *ATHB*, and *SAUR23*, known as downstream targets, was also found to be lower in the ANT samples than in the LAB samples. In addition, the expression of *PIF4* ortholog was dependent on temperature, and the phenotype we observed was in agreement with Kumar *et al*.^36^.

Phenylpropanoid biosynthesis pathway produces a wide variety of secondary metabolites which contribute to all aspects of plant responses towards biotic and abiotic stimuli, with lignins and flavanones being representative products^30^. In this study, almost all genes involved in the ‘phenylpropanoid biosynthesis’ pathway were strongly upregulated in ANT samples implying that products and its intermediates could have crucial roles to protect plant cells from abiotic and biotic stress. In *Arabidopsis*, *Zea mays*, *Eucalyptus globulus*, *Lotus japonicus* and *Miscanthus*, the major enzymes of the phenylpropanoid pathway such as PAL, CCR, CAD, CHS and CHI have been shown to be strongly induced by cold, drought, UV irradiation or pathogen infection but the gene expression patterns are different according to tissue- and species-specific^33,49–54^. In this regard, transcriptional up-regulation of phenylpropanoid biosynthesis genes in ANT samples may be the result of cross-talk responses due to various environmental factors such as cold, drought and pathogenesis, and this result is correlated with the observation that *C. quitensis* plants grown in open areas with relatively low ambient temperature exhibited a higher fraction of cell wall chemical components (hemicellulose, cellulose and lignin content) than plants grown inside OTC^22^.

Overall DEG analyses revealed that the transcriptome of field plants of *C. quitensis* has major changes in ‘photosynthesis’ genes and ‘stress response’ genes to adapt to an extreme environment. These results can be compared with transcriptome results from another extremophyte, *Eutrema salsugineum*, which has a very clear phenotypic plasticity in natural habitats vs. controlled chambers^55,56^. The transcriptome studies of this species have also shown that stress response genes and photosynthetic genes are correlated with phenotypic plasticity at field conditions^56^, suggesting that there may be a common mechanism for how extremophytes adapt to the environment.

In Antarctic *C. quitensis*, about 40% of PCESR orthologs (14 of the 35 genes), which are known to be induced early in response to abiotic stress in *Arabidopsis*, were expressed at high levels. In our *in-silico* study and qPCR expression assay, we identified 14 PCESR genes with diverse functions that were highly expressed in ANT samples and induced by abiotic stresses. The stress response is initiated by the intracellular Ca^2+^ change and the generation of ROS^3,57^. This intracellular signaling induces the expression of transcriptional regulatory elements, which in turn induce the expression of downstream genes by binding to *cis*-elements in the regulatory regions^3,57^. Among the 14 genes, calmodulin-like proteins and transcription factor genes such as AP2/ERF, NAC, and MYB were included. C2H2 zinc finger proteins ZAT10 and ZAT12 maintained high levels of expression in ANT samples and were specifically induced by low-temperature^58,59^. ZAT12 is activated by different abiotic stresses and is required for systemic H_2_O_2_ signaling^58^. Another C2H2 zinc finger protein, ZAT10, is known to be systemically regulated and induced by different stresses and essential for priming for systemic acquired acclimation^59^. We have confirmed that orthologs of ZAT10 and ZAT12 are expressed at high levels in ANT samples of *C. quitensis* experiencing various environmental stresses and presume that the expression of these genes will help maintain intracellular homeostasis in response to environmental stress. In addition, an E3-ligase, ring finger proteins, a PGIP protein and various kinds of transporter proteins, which have been reported to be involved in modulation of abiotic stress responses by posttranslational modification, inhibiting of ice-recrystallization or transport of metabolites^47,60,61^, were also highly expressed in ANT samples, and expression was induced by cold stress as well. It should be noted that we selected genes that are known to be commonly expressed in the early stages of various stress reactions, and we found that they have higher levels of transcripts in ANT samples that have undergone environmental stress such as low-temperature, high salt, drought, or high UV. However, when we stressed plants individually in the laboratory, many genes were induced by low-temperatures, but not by high salt or dehydration. We cannot completely exclude the possibility that we did not see the earlier reaction by setting a single time point after 24 h in our experiment. However, we would expect that the main cause of gene induction by abiotic stress in field sample is low-temperature.

In this study, we found that multiple adaptations such as modulation of energy metabolism, change in PCESR gene expression pattern, and morphological adaptions are present in *C. quitensis* through a global analysis of field transcriptome of *C. quitensis*. The results suggest that plants adapted to the extreme environment have developed a modified genetic control system as a survival strategy against harsh conditions. Thus, the *C. quitensis* transcriptome profile broadens our understanding of how plants tolerate extreme environments and their adaptive responses to climate change.

## Methods

### Study area and sampling in the Antarctic field

*C. quitensis* plants growing under field conditions were collected in the vicinity of the Korean King Sejong Antarctic Station (62°14′29″ S; 58°44′18″ W), on the Barton Peninsula of King George Island on January 2013. The samples were immediately ground in TRIzol reagent (Invitrogen, Carlsbad, CA, USA) at the sampling site and transferred in 1 h to the laboratory in King Sejong Antarctic Station for RNA extraction and designated as ANT samples. Photosynthetic active radiation (PAR) and air temperatures at Barton Peninsula were measured during January (2013) with a data logger CR800 (Campbell Scientific, Logan, UT, USA) connected with thermocouples and PAR sensors. Microclimatic conditions on Barton Peninsula during January are shown in Fig. 1A. The air temperature of the site was measured from a temperature sensor installed 5 cm above the soil surface. During the warm months, temperatures dropped below 0 °C for only a few days, and the maximum temperature was 14 °C. The average monthly temperature was ca. 2.7 °C. Maximum PAR intensity varied from ca. 1,845 μmol m^−1^ s^−1^ on a clear day (19^th^ January 2013) to ca. 629 μmol m^−1^ s^−1^ on a cloudy day (15^th^ January 2013). The luminosity was close to zero from midnight to 04:00 AM. We harvested ANT samples between 12:00 ~ 14:00 local time.

### Growth conditions of laboratory plants

Some plants from Barton Peninsula were transferred to the laboratory in Korea Polar Research Institute and have been grown hydroponically supplemented with 0.5 × Murashige and Skoog (MS) medium containing 2% sucrose in a climate chamber under a prolonged long day (20:4 h light: dark) cycle with a light intensity of 150 μmol m^−2^ s^−1^ at 16 °C. After growing plants for three weeks in a climate chamber, plants with horizontal diameters of 1.5 ~ 2 cm in similar developmental stages were collected. The old leaves of the lower part of the plant were removed, and the newly developed leaves of the upper part were sampled for RNA extraction and designated as LAB samples. For cold stress treatments, plants grown at 16 °C with 1.5 ~ 2 cm horizontal diameter were transferred to 2 °C and incubated until harvest. For salt stress treatments, plants were transferred onto 0.5 × MS liquid medium, grown for 7 days and then transferred onto the 0.5 × MS liquid medium supplemented with 150 mM NaCl^62^. For drought stress treatments, plants were transferred onto 3 mm filter paper, exposed to air in a clean bench for 30 min. Subsequently, the plants on filter papers were transferred back to the empty growth boxes and cultivated until harvest^32^. Plants were harvested for RNA extraction after 0, 1 and 7 days under each condition and prepared in three different biological replicates. All lab−cultured samples were harvested 8 hours after lights-on.

### Leaf length measurement

To compare morphological differences depending on temperature, plants grown at 16 °C with 1.5 ~ 2 cm in horizontal diameter were transferred to growth chambers at 2 °C and 8 °C and cultured for 4 weeks. The length of individual leaves was measured by ImageJ programs (https://imagej.nih.gov/ij/). All plants for measurements were prepared with five biological replicates. Statistical analysis was performed by student’s *t*-test (*p* < 0.05).

### RNA extraction and RNA-Seq library construction

Total RNAs were extracted using TRIzol reagent, treated with DNase I (QIAGEN, Hilden, Germany) to remove contaminant genomic DNA, and were subsequently purified using the RNeasy mini kit (QIAGEN) following manufacturer’s protocols. Three biological replicates were prepared. RNA integrity and concentration were determined using a Bioanalyzer (RIN > 6) (Agilent Technologies, Waldbronn, Germany) and a Qubit®RNA Broad-range Assay Kit (Thermo Fisher Scientific, Waltham, MA, USA), respectively. To construct RNA-Seq libraries, 1.5 µg of total RNA from each sample was used as input for the TruSeq RNA sample prep kit v2 (Illumina, San Diego, CA, USA). The libraries were quantified using a Bioanalyzer and the library qPCR quantification method following the Illumina guideline. After quantification, they were multiplexed in equal ratios and loaded onto a single flow cell of Illumina HiSeq Rapid SBS kit v2 (2 × 100 runs). Sequencing was performed on a HiSeq. 2500 Sequencer system (Illumina) and a total of 16.2 Gb (160M paired-end reads) of sequencing data were generated (Q30 > 93%).

### *De novo* assembly and annotation

*De novo* assembly was performed using the CLC Genomics Workbench v7.5 software (QIAGEN). The reads were filtered by trimming adapter sequences, excluding low-quality sequences (quality score <0.001, ambiguity <2 bps) and removing too short sequences (length >50 bps) and duplicates. The resulting reads were assembled with following parameters (word size = 20 and bubble size = 50, length >200 bps), and then the assembled contigs were clustered using CD-HIT^63^. A total of 95,010 assembled contigs were subjected to BLASTX searches against the non-redundant protein database with an E-value threshold of 1 × 10^−5^. Gene ontology (GO) mapping and annotation were performed with an annotation cutoff of E-value < 1 × 10^−10^ and using Blast2GO platform^64^. GO enrichment analysis was performed with using AgriGO^65^ with Fisher’s exact test (FDR < 0.05). Putative full-length cDNAs were predicted by comparison of BLASTX reports from the UniProt databases with a web-based ORF prediction tool, Full-lengther^66^. To identify orthologs, translated sequences of *C. quitensis* derived from ORF prediction and the *Arabidopsis* protein sequence datasets (TAIR version 10.0) which were obtained from the JGI Phytozome website (https://phytozome.jgi.doe.gov) were compared. The orthologs were identified by reciprocal best-hit analysis for selecting BLASTP parameters with options of soft masking and Smith-Waterman alignment (-seg yes -soft_masking true -use_sw_tback), lowest e-value, query coverage >50 and protein identity >50 as best hits^67^. To map the contigs to the metabolic pathways, the translated sequences of contigs were blasted to the pathways of Kyoto Encyclopedia of Genes and Genomes (KEGG) databases using internal annotation tool in KEGG website^25^. KEGG enrichment analysis was performed using a KOBAS web server^68^.

### Differentially expressed gene analysis

The expression values were measured in fragments per kilobase of exon model per million mapped reads (FPKM) normalized values^69^. For statistical analysis, Baggerley’s tests^70^ and *t*-tests were performed on the normalized read counts. In addition, several relevant values for analysis, such as *p*-values and corrected *p*-values for multiple corrections, were calculated in the “two-group comparison” option. Through the statistical analysis, the differentially expressed genes were determined using a cut-off value (corrected *p*-value of false discovery rate <0.05 and difference ≠ 0).

### qPCR analysis

Total RNA was extracted from leaves of plant samples and purified using the RNeasy Plant Mini Kit (QIAGEN) as described previously. cDNA was synthesized from 2 ug total RNA extracted from samples using Superscript III (Invitrogen). Gene-specific primers were designed according to the sequences of the contigs and are listed in Supplementary Table S12. To select internal control genes, we selected 7 candidate genes which have steady expressions at both LAB and ANT transcriptome data as follows: *18S rRNA* (contig32901), *UBC28*, ubiquitin-conjugating enzyme E2 28-like (contig3602), *RPB6A*, DNA-directed RNA polymerase subunit (contig9755), *TIM*, triosephosphate chloroplast-like (contig19814), *CHC*, clathrin heavy chain (contig6535), *GLX2-4*, lactoylglutathione chloroplast-like (contig18505), *RPL3*, 50S ribosomal protein L3 (conti39479). To validate the fitness of the reference genes, we performed the amplification efficiency test and the gene stability test by RefFinder (http://leonxie.esy.es/RefFinder/)^71^. The amplification efficiency between 95–105% (R^2^ > 0.97) was determined as good. And RefFinder analysis carried out using Ct values obtained from all experimental samples. As results of both analyses, ranking for reference gene was followed: *TIM* > *18S* > *GLX2-4* > *UBC28* > *RPL3* > *RPB6A* > *CHC*. Also, geNorm algorism^72^ gave a suggestion for a combination of *TIM* and *18S* genes, and thus we used them as references genes. The primer information is listed in Supplementary Table S12. A qPCR was performed with biological triplicates using SYBR® Premix Ex Taq™ DNA polymerase (Takara Bio Inc., Shiga, Japan) and the Mx3000P Real-Time PCR System (Stratagene, La Jolla, CA, USA).

### Ethics approval and consent to participate

This study including sample collection and experimental research conducted on these materials was according to the law on activities and environmental protection to Antarctic approved by the Minister of Foreign Affairs and Trade of the Republic of Korea.

### Data availability

The Illumina raw sequencing data and the sequence information of assembled contigs are available at https://www.ncbi.nlm.nih.gov/bioproject/PRJNA388703, [NCBI bioproject ID: PRJNA388703, Sequence Read Archive (SRA) ID: SRX2913822 and SRX2913823].

## Electronic supplementary material

Supplementary Information

Dataset
