# Supplementary material for: Comparative transcriptome analysis of field- and chamber-grown samples of Colobanthus quitensis (Kunth) Bartl, an Antarctic flowering plant
Source: Sci Rep. 2018 Jul 23;8:11049. doi: 10.1038/s41598-018-29335-4 (PMC6056519; doi:10.1038/s41598-018-29335-4)
Supplement: Supplementary file 1 — Supplementary Information [file 41598_2018_29335_MOESM1_ESM.pdf]

Supplementary Information

Comparative transcriptome analysis of field- and chamber-grown samples of *Colobanthus quitensis* (Kunth) Bartl, an Antarctic flowering plant

Sung Mi Cho[1], Hyeongseok Lee[1,2], Hojin Jo[1,2], Horim Lee[3], Yoonjee Kang[1], Hyun Park[1,2] and Jungeun Lee[1,2*]

[1]Unit of Polar genomics, Korea Polar Research Institute, KIOST, Incheon 21990, Republic of Korea

[2]Department of Polar Sciences, University of Science and Technology, Incheon 21990, Republic of Korea

[3] Department of Biotechnology, Duksung Women's University, Seoul 01369, Republic of Korea

[*]To whom correspondence should be addressed:
Dr. Jungeun Lee, Unit of Polar genomics, Korea Polar Research Institute, KIOST, Incheon 21990, Republic of Korea; Tel: +82-32-760-5576; Fax: +82-32-760-5509; E-mail: jelee@kopri.re.kr

Supplementary Figures

Supplementary Fig S1. The species distribution of blast matches to the UniProt plant databases

Supplementary Fig S2. Functional GO classification of the *C. quitensis* transcriptome

Supplementary Fig S3. Pathway assignment based on KEGG

Supplementary Fig S4. A Venn diagram showing the count of orthologs shared between *C. quitensis* proteome (Uniprot - predicted) and *A. thaliana* proteome (TAIR10)

Supplementary Tables (Excel format)

Supplementary Table S1. Summary of sequencing, assembly, and annotation

Supplementary Table S2. Gene ontology classification unigenes using the PlantGO slim terms

Supplementary Table S3. KEGG pathway mapping of total contigs of *C. quitensis*

Supplementary Table S4. Statistics of SSR markers identified from *C. quitensis* transcriptome analysis

Supplementary Table S5. List of upregulated genes in ANT vs. LAB samples

Supplementary Table S6. List of downregulated genes in ANT vs. LAB samples

Supplementary Table S7. The result of GO enrichment test for upregulated DEGs

Supplementary Table S8. The result of GO enrichment test for downregulated DEGs

Supplementary Table S9. The result of KEGG pathway enrichment test for upregulated DEGs

Supplementary Table S10. The result of KEGG pathway enrichment test for downregulated DEGs

Supplementary Table S11. List of 8,530 orthologs identified from the reciprocal best hit analysis between *C. quitensis* contigs and the *A. thaliana* gene dataset

Supplementary Table S12. Primer sequences used for qPCR analysis

Supplementary Figures

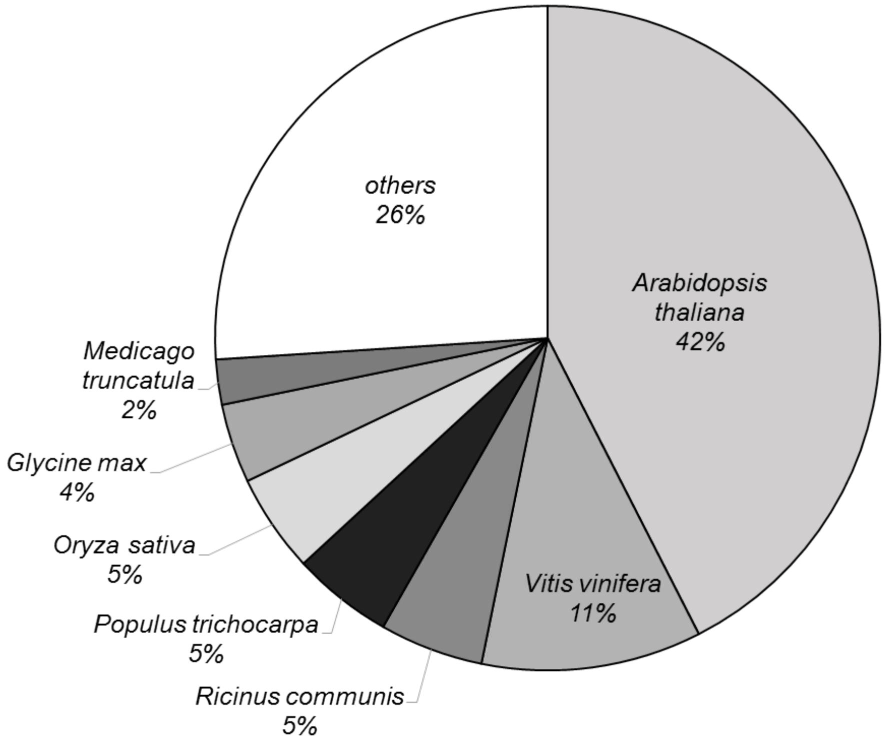

Supplementary Fig S1. The species distribution of blast matches to the UniProt plant databases

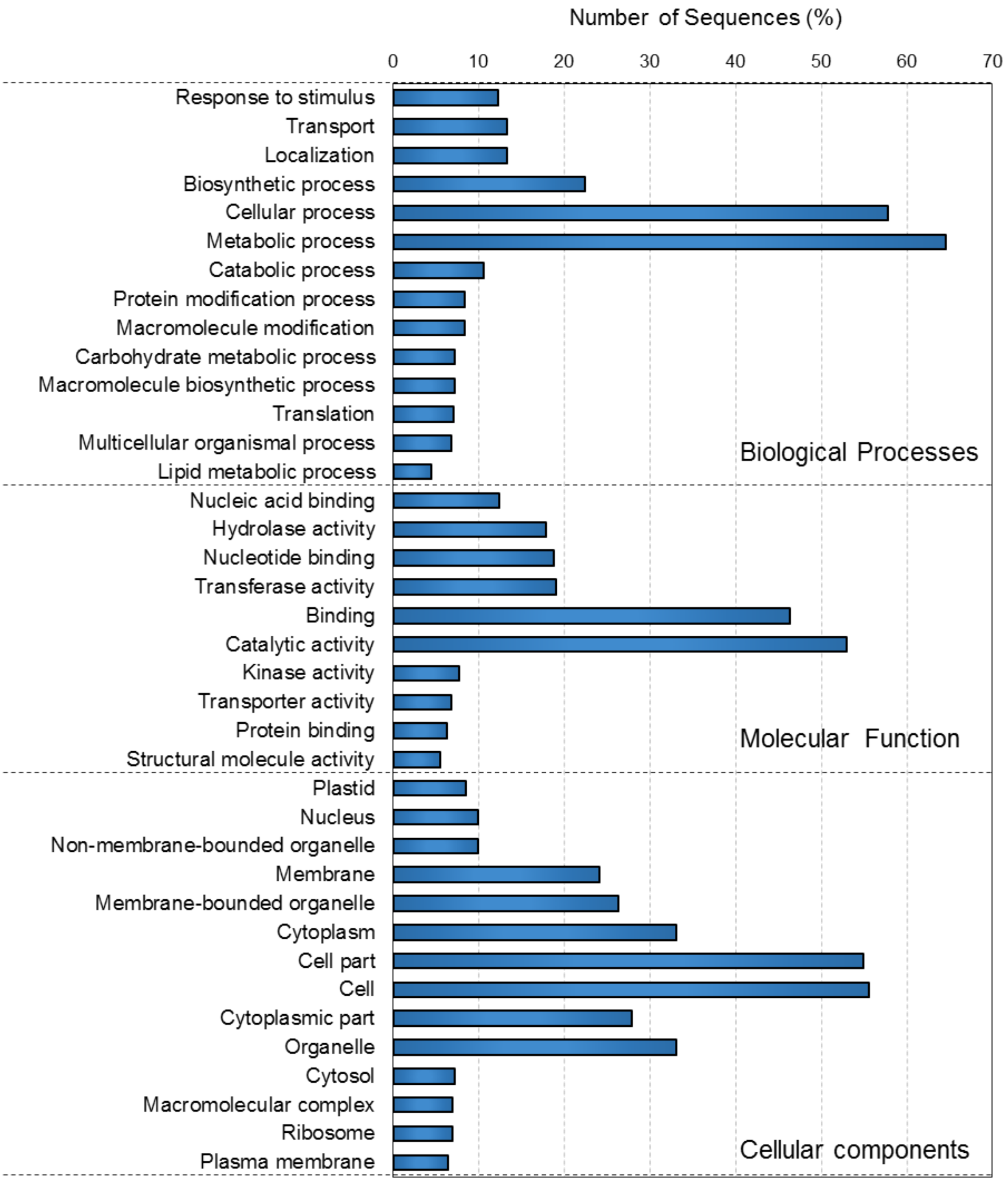

Supplementary Fig S2. Functional GO classification of the *C. quitensis* transcriptome. Sequences with BLASTX matches were assigned GO terms and classified into different functional categories (biological process, molecular function, and cellular component).

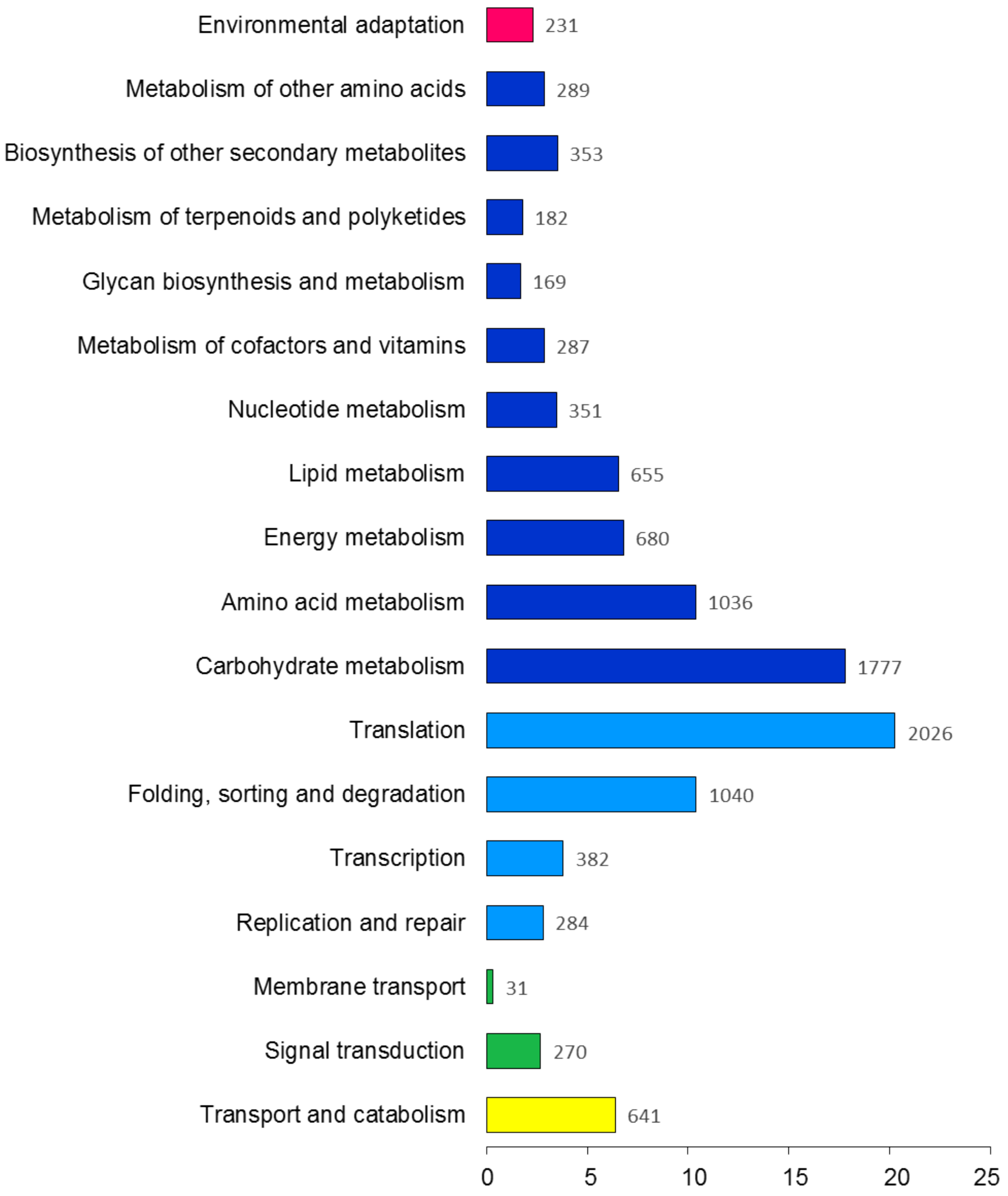

Supplementary Fig S3. Pathway assignment based on KEGG. Pathways were assigned into five categories. OS: Organismal systems, M: Metabolism, GIP: Genetic information processing, EIP: Environmental information processing, CP: Cellular processes

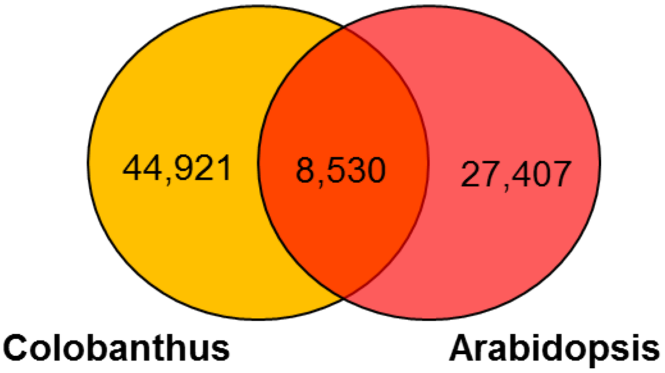

Supplementary Fig S4. A Venn diagram showing the count of orthologs shared between *C. quitensis* proteome (Uniprot - predicted) and *A. thaliana* proteome (TAIR10). Orthologous sequences between the two species were determined using the reciprocal best blast hit method (Moreno-Hagelsieb and Latimer 2008).
